# In vitro digestion and culture in Caco‐2 cells to assess the bioavailability of fatty acids: A case study in meat matrix enriched with ω‐3 microcapsules

**DOI:** 10.1002/fsn3.4241

**Published:** 2024-06-17

**Authors:** Juan Carlos Solomando, Teresa Antequera, Matías Estaras, Antonio González, Trinidad Perez‐Palacios

**Affiliations:** ^1^ Research Institute of Meat and Meat Products (IProCar) University of Extremadura Cáceres Spain; ^2^ Department of Physiology, Institute of Biomarkers and Molecular Pathologies University of Extremadura Cáceres Spain

**Keywords:** bioaccessibility, bioavailability, Caco‐2 cell, fatty acids, fish oil microcapsule, meat product

## Abstract

This work aimed to evaluate the Caco‐2 cells as a model to study the epithelial transport of intestinal lipid extracts subjected to in vitro digestion, to establish a standard protocol for the determination of bioaccessibility and bioavailability of fatty acids in meat matrix, especially in those enriched with ω‐3 (eicosapentaenoic (EPA) and docosahexaenoic acids (DHA)). Samples were first subjected to in vitro digestion, and then, the intestinal extract was incubated with Caco‐2 cells. A first trial was conducted to select the most influencing variables on the fatty acid transport during Caco‐2 cell incubation: fat quantity on the intestinal extract, incubation time, and shaking. Then, a second experiment was carried out to determine the influence of these variables, being the fat quantity and the incubation time the most influencing factors on the transport and bioavailability of fatty acids. The effect of shaking was not so marked but seemed to improve the bioavailability of saturated fatty acids. This study also allows establishing the most suitable conditions: intestinal extracts with 30 mg of fat, longer incubation times (8 h), and shaking, achieving active and passive fatty acid transport without compromising the integrity of the Caco‐2 cell monolayer. The accurate results obtained for major and minor fatty acids, especially EPA and DHA are remarkable, due to the interest in these bioactive compounds. Thus, this study provides a combined protocol based on static in vitro digestion followed by Caco‐2 cell incubation to assess the bioaccessibility and bioavailability of fatty acids in meat samples.

## Graphical abstract

Bioaccessibility and bioavailability of meat products have been first described. Fat quantity, incubation time, and shake affected the bioavailability. A protocol for in vitro digestion and Caco‐2 cell incubation was set for meat samples.

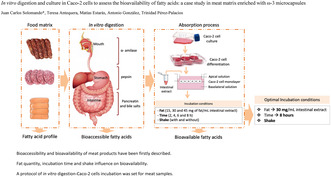

## 1 INTRODUCTION

Bioaccessibility (BAC) is the fraction of an ingested component that is accessible in the intestine to be absorbed through the epithelial cells (Fernández‐García et al., 2009) and it should be assessed by in vitro gastrointestinal digestion. In comparison to in vivo assays, the in vitro trials are cheaper, need fewer supplies, and do not have ethical questions; these aspects have led to this technique being highly applied in food and nutrition experiments (Dahan & Hoffman, 2006; Kaukonen et al., 2004). To accurately determine the BAC, the in vitro procedures have to reproduce faithfully gastrointestinal conditions. However, there are many factors (enzyme concentration, pH, time, mineral composition of digestive fluids, etc.) that can substantially vary the obtained results (Porter et al., 2004; Salsinha et al., 2023; Sek et al., 2002). In trying to solve this drawback, a standardized in vitro gastrointestinal digestion protocol has been proposed (Minekus et al., 2014).

On the other hand, the term bioavailability (BVB) refers to the fraction of a component that is to the systemic circulation (Holst & Williamson, 2008). In the case of the lipids, they are emulsified and hydrolyzed to produce 2‐monoacylglycerols (2‐MAG) and free fatty acids (FFA) in micelles (Ho & Storch, 2001; Salsinha et al., 2023), which are absorbed through the epithelial cells (in a monolayer) of the intestinal mucosa to finally reach the lumen. To carry out an in vitro intestinal absorption of bioaccessible lipid hydrolysates, Caco‐2 cells (from human colon carcinoma) have been preferably used because they express many phenotypic characteristics of absorbing mature enterocytes, such as cell polarity, binding complexes, brush border, specific digestive enzymes, and transport processes (Beguin et al., 2014; Tsuzuki, 2007), and are considered as an appropriate model to reproduce the transcellular traffic of physiological events related to the bioavailability of different intestinal bioaccessible compounds (Parada & Aguilera, 2007).

In this sense, this cell line has been used successfully in previous studies to evaluate the in vitro bioavailability of antioxidants (Bhushani et al., 2017; Parada & Aguilera, 2007), drugs (Keemink & Bergström, 2018; Van Breemen & Li, 2005), FFA and 2‐MAG (Buhrke et al., 2012; Tsuzuki, 2007), and minerals (Frontela et al., 2009), collecting the compound resulting after passing through the monolayer of Caco‐2 cells as the final bioavailable amount. The Caco‐2 model of Tsuzuki (2007) to investigate dietary lipid absorption indicated the preparation of mixed micelles, including FFA, 2‐MAG, bile salts, and lysophospholipids, to be incubated with a cell monolayer. However, this procedure did not reproduce the whole lipid digestion process, only considering the final stage of culture in Caco‐2 cells but not the previous gastrointestinal digestion, and it was carried out with lipid standards but not digested extracts.

When making the choice of the type of food to be fortified with functional ingredients, the demands and requirements of the market should be considered. Thus, enriched food products should have good acceptability, high intake, and moderate price. Besides, the current widespread intake of ready‐to‐eat foods, due to the increasing lack of time for traditional cooking, should be also accounted (OMS, 2015). In this sense, meat products can be a good option to be enriched in ω‐3 fatty acids because, in general, they are well accepted and highly consumed (around 3–4 times per week), there is a wide variety, and many of them do not have a high price. Besides, their lipid profile is sometimes questioned, which is highly related to the quantity of saturated and polyunsaturated fatty acids (SFA and PUFA, respectively) that are moderate–high and low, respectively (Nuernberg et al., 2005). In fact, different strategies have been evaluated to increase the ω‐3 fatty acid content in this type of food: the direct inclusion of fish oil to feedings for animals or even to meat products, and the addition of a fish oil emulsion to the meat products, which not only get the enrichment of ω‐3 fatty acids but also have a negative impact on oxidative stability and some sensory characteristics, even in meat products with antioxidant additives (Bolger et al., 2017; Lee et al., 2006; Valencia et al., 2007). More recently, the strategy based on the microencapsulation technique has also been evaluated; the addition of fish oil microcapsules to meat products has achieved to rise in the EPA and DHA quantity in meat products and no marked negative effects on the proximal composition, oxidative stability, or sensory profile (Solomando et al., 2021; Solomando et al., 2020a).

Quantification of fatty acids is crucial to guarantee that added food has been actually enriched and enables the food labeling as a “source of ω‐3 fatty acids” or “high content of ω‐3 fatty acids” (EU, 2010). However, it does not assure their release and/or absorption at intestinal level because of the influence of different issues of the gastrointestinal process (i.e., food component interactions, recognition by digestive enzymes, enterocyte transport, or absorption on bioaccessibility and bioavailability (Tsuzuki, 2007)). The bioaccessibility of EPA and DHA in meat products added with ω‐3 microcapsules has been determined using in vitro digestion (Solomando et al., 2020a, 2020c), however, among the scientific literature in meat products, protocols or studies about the bioavailability of fatty acids have not been found.

Considering these premises, this study aimed to evaluate the viability of Caco‐2 cells as a model to study the epithelial transport of gastrointestinal lipid extracts from treated meat samples, with special interest in that enriched with ω‐3 fatty acids and with the objective to develop an in vitro gastrointestinal method to determine the bioaccessibility and bioavailability of fatty acids in meat matrix.

## 2 MATERIALS AND METHODS

### 2.1 Experimental design

A first trial was conducted following previous procedures with Caco‐2 cells for absorption experiments (Beguin et al., 2014; Tsuzuki, 2007; Van Breemen & Li, 2005). Pork burgers and pork dry‐cured sausages were purchased in a local market and analyzed by percentage of total lipids and fatty acid composition and subjected to in vitro digestion and subsequent culture with Caco‐2 cells to calculate the BAC and BVB of fatty acids. There were triplicate of experimental samples (*n* = 3) and all analyses were done in triplicate. Pork burgers were made with Iberian ham meat, water, salt, vegetable fiber, spices, spice extracts, and antioxidants (E‐316) with the following nutritional composition, expressed per 100 g of product: 904 Kj/218 Kcal of energy value; 16 g of fat, of which 5.7 g were saturated; <0.5 g of carbohydrates, of which <0.5 g were sugars; 0.8 g of fiber; 18 g of protein; and 1.5 g of salt. Pork dry‐cured sausages were made with Iberian pork meat, fat, salt, dextrose, soy protein, spices, stabilizers (E‐450 and E‐451), antioxidant (E‐301), preservatives (E‐250 and E‐252), flavor enhancer (E‐621), and coloring (E‐120) with the following nutritional composition, expressed per 100 g of product: 2243 Kj/542 Kcal of energy value; 38.4 g of fat, of which 19.06 g were saturated; 1.7 g carbohydrates, of which 0.7 g were sugars; 22.7 g of protein; and 3.8 g of salt.

**FIGURE 1:**
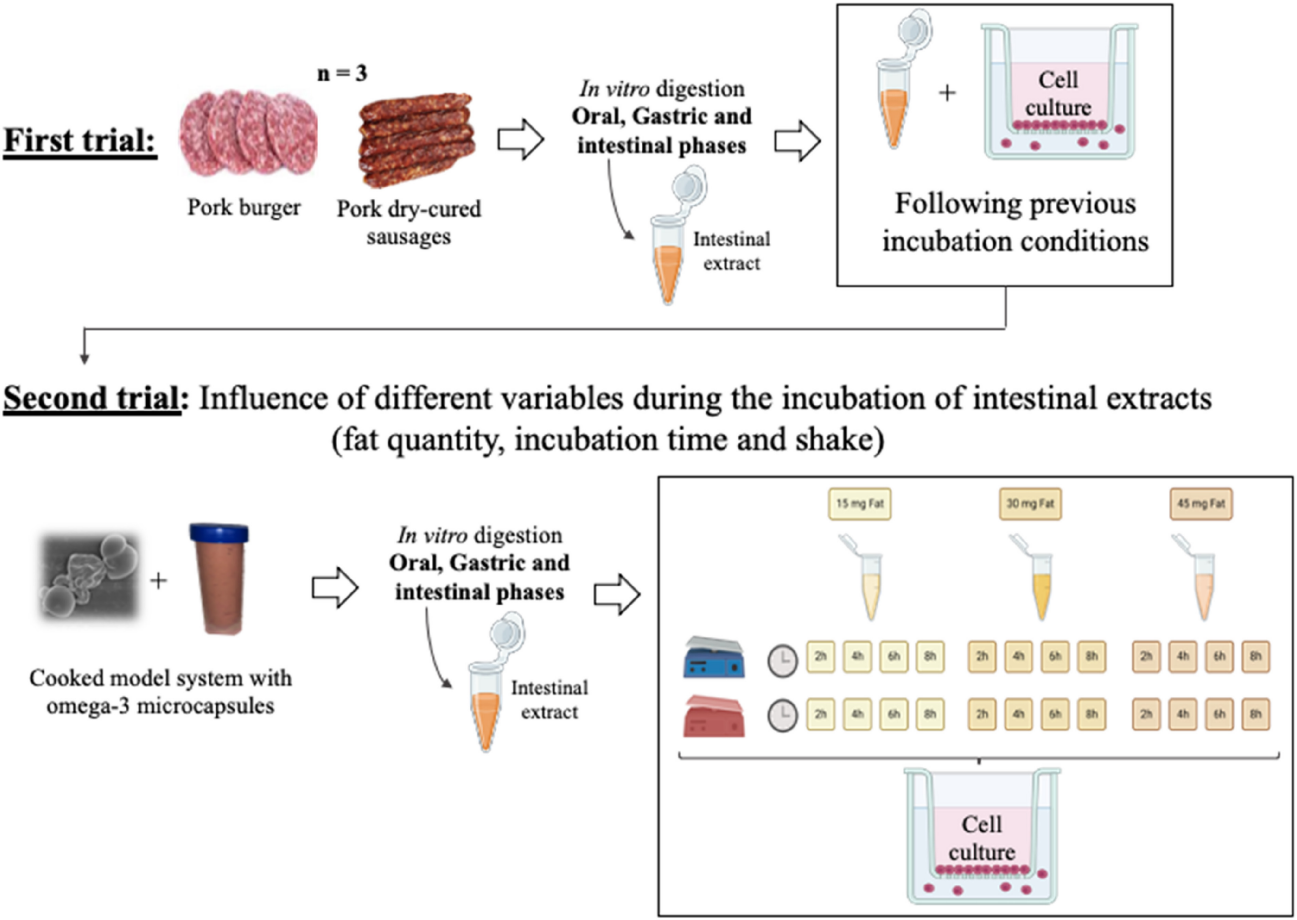
Summary of experimental design section.

Results obtained from this first trial led us to evaluate the influence of different variables during the incubation of intestinal extracts obtained from meat matrix enriched with fish oil microcapsules with Caco‐2 cells, to finally achieve an optimized protocol for determining the bioavailability of fatty acids. For that, a cooked meat model system enriched with ω‐3 microcapsules was prepared. In these samples, the percentage of total lipids and fatty acid composition were also analyzed and subjected to in vitro digestion. The intestinal extract was incubated with Caco‐2 cells, varying fat quantity (15, 30, and 45 mg of fat/mL intestinal extract), incubation time (2, 4, 6, and 8 h) at 37°C in an incubator with 5% CO_2_ and 95% relative humidity, and shaking (with and without), having a total of 24 combinations. For each combination, samples were prepared and analyzed in triplicate. Figure 1 shows a summary of the experimental design.

### 2.2 Biological materials and reagents

Fish oil (Biomega Nutrition, Galicia, Spain), soybean lecithin (Across Organics, Madrid, Spain), chitosan with 95% of deacetylation (Trades, Murcia, Spain), maltodextrin with 12% DE (Roquette, Lestrem, France), and food‐grade acetic acid (Scharlau, Barcelona, Spain) were used for the preparation of the emulsions. Dry‐cured sausages and pork burgers were purchased from a local grocery and cooked model system were elaborated with pork meat, water, pork fat and salt. α‐Amylase type IX‐A, 1000–3000/U/mg protein (EC 3.2.1.1), pepsin from porcine gastric mucosa 3200–4500 U/mg protein (EC 3.4.23.1), porcine pancreatic lipase 350 U/mg protein (EC 3.1.1.3), and porcine bile extract (EC 232.369) (Sigma‐Aldrich, Madrid, Spain) were used for the simulated digestion. NaCl, KCl, KH_2_PO_4_, NaHCO₃, MgCl_2_, (NH_4_)_2_CO_3_, and CaCl_2_ (Scharlau) were used as electrolytes in simulated salivary, gastric, and intestinal fluids, and NaOH and HCl (Scharlau) to adjust the pH. Caco‐2 cells were obtained from the European Collection of Cell Cultures (ECACC; number 86010202, Salisbury, UK). Minimum essential medium (MEM), fetal bovine serum (FBS), nonessential amino acids, L‐glutamine, and penicillin–streptomycin (Sigma‐Aldrich, Madrid, Spain) were used for culture and grew in 75 cm^2^ Nunc flasks and polycarbonate membrane chamber inserts (3.14 cm^2^ diameter, 0.4 μm pore size; Transwell Costar Corp.) (ThermoFisher, Waltham, EE. UU). Trypsin–EDTA solution (Sigma‐Aldrich, Madrid, Spain) was used for cell subculture. Phosphate‐buffered saline (PBS) and Hank's balanced salt solution (HBSS) (Sigma‐Aldrich) were used as washing solutions. Chloroform, methanol, and sodium sulfate (Scharlau) were used for fat extraction. Sulfuric acid, methanol, sodium metal, and hexane 96% (Scharlau) were used for the fatty acids transesterification.

### 2.3 Preparation of fish oil microcapsules

Microcapsules were prepared following the methodology of Solomando, Antequera, Ruiz‐Carrascal, and Perez‐Palacios (2020). Fish oil (20 g) and lecithin (6 g) were mixed with a magnetic stirrer. Then, water was added until a total weight of 200 g was achieved and homogenized (1120 *g*, 10 min) using an Ultraturrax T‐18 basic (IKA, Germany). In this way, the primary emulsion was obtained and then homogenized at high pressure (SPX, Model APV‐200a, Silkeborg, Denmark) under the previously optimized conditions (1100 Ba and 2 passes). The homogenized primary emulsion was blended with 200 g of 1% chitosan (w/w) in acetic acid 1% by slow agitation for 15 min. The final step consists of adding 400 g of maltodextrin solution (120 g maltodextrin + 280 g water), and the feed emulsions (800 g) were dried in a laboratory‐scale spray drier equipped with a 0.5 mm nozzle (Mini spray‐dryer B‐290, Buchi, Switzerland). The emulsions were constantly agitated in a magnetic stirrer during spray‐drying process. The aspirator rate was adjusted at 80%, the feed rate was 1 L/h, and the inlet and outlet temperatures were set at 180 and 90°C, respectively. The collected dried powders were stored in containers at 4°C.

### 2.4 Elaboration of cooked model system

The cooked model system was made with minced pork (65%), minced pork belly (15%), water (18%), salt (2%), and 10.6 g of multilayered fish oil microcapsules per 100 g of dough. The quantity of microcapsules added was calculated to excess the required abundance of EPA + DHA to label the food as “high in ω‐3 fatty acids”: at least 80 mg of the sum of EPA and DHA per 100 g and per 100 Kcal (EU, 2010). The mixture was kneaded by using a Thermomix (Wuppertal, Germany), stuffed into 50 mL Falcon tubes, and heated in a water bath at 80°C for 15 min.

### 2.5 Simulated digestion

The release of fatty acids from the meat products was evaluated in simulated digestion conditions, including three sequential steps: oral, gastric, and intestinal. Stock solutions, simulated salivary, gastric, and intestinal fluids (SSF, SGF, and SIF, respectively) were measured according to the methodology of Minekus et al. (2014). Salivary α‐amylase was prepared in SSF to a final concentration of 75 U/mL, pepsin in SGF to a final concentration of 2000 U/mL, as well as pancreatin and bile salts in SIF to a final concentration of 100 U/mL and 10 mM, respectively. Minced fresh matter of all meat products (5 g) was weighed in a falcon tube and mixed with 3.5 mL of SSF, 0.5 mL of salivary α‐amylase solution, 25 μL of 0.3 mol/L CaCl_2_, and 975 μL of distilled water. The mixture was homogenized by vortex for 1 min and stirred at 300 rpm for 2 min at 37°C. Gastric digestion was continued by immediate addition of 7.5 mL of SGF, 1.6 mL of pepsin, 5 μL of 0.3 mol/L CaCl_2_, and 0.278 mL of distilled water to the oral bolus, and pH was adjusted to 3.0 with 0.2 mL of HCl 6 M. The mixture was incubated at 37°C with shaking at 300 rpm during 2 h. Then, intestinal digestion was followed by the addition of 11 mL of SIF, 5 mL of pancreatin, 2.5 mL of bile salts, 0.3 mL of pancreatic lipase, and 40 μL of CaCl_2_ 0.3 M, and pH was adjusted to 7 with 150 μL of NaOH 1 M. Distilled water (1.31 mL) was added and kept under agitation (120 rpm) at 37°C for 2 h. At the end of the in vitro digestion, all samples were frozen with liquid nitrogen until analysis. The fat was extracted from the intestinal extract with hexane (5 mL), centrifuged (4480 *g*, 20 min), and filtrated through filters with a pore size of 0.2 μm to quantify the BAC fat.

### 2.6 Cell culture

The Caco‐2 cells used in this work were at passages 37–54 and cultivated in 75 cm^2^ Nunc flasks containing MEM supplemented with 10% v/v heat‐inactivated FBS, 1% v/v nonessential amino acids, 1% v/v l‐glutamine, and 1% v/v antibiotic solution (penicillin–streptomycin) at 37°C in an incubator with 5% CO_2_ and 95% air atmosphere. The growth medium was replenished every 2–3 days. For the experiment, cells were seeded onto polycarbonate membrane chamber inserts (3.14 cm^2^, 0.4 μm pore size; Transwell Costar Corp.) at a density of 50,000 cells/cm^2^ and allowed to differentiate on filters for 21 days, replenishing the growth medium every 2–3 days and checking the integrity by measure of transepithelial electrical resistance (TEER) (Sambuy et al., 2005). Before each experiment, the growth medium was removed, and apical and basolateral cell surfaces were washed three times with PBS at 37°C. Then, 1 mL of intestinal extract and 1.5 mL of MEM supplemented (pH 7.4) were added to the apical chamber of each cell monolayer, respectively. In the first trial with commercial pork burger and dry‐cured sausage, the intestinal extract was incubated for 4 h without shaking with 5% CO_2_ and 95% relative humidity, following the method of Salsinha et al. (2023) with slight modifications. After incubation process, samples from the apical, basal, and membrane compartments were collected, transferred to glass tubes, and fat was extracted with hexane to determine the BVB by acid transesterification and subsequent analysis by gas chromatography following the methodology described in Section 2.8 of this work. In the case of the trial to optimize incubation conditions with the intestinal extract from cooked meat model system enriched with ω‐3 source, different fat quantities (15, 30, and 45 mg of fat/mL intestinal extract), incubation time (2, 4, 6, and 8 h) and shaking (with and without) were evaluated. It was carried out at 37°C in an incubator MR‐12 Rocker‐Shaker (Biosan, Almería, Spain) with 5% CO_2_ and 95% relative humidity, at a speed of 40 rpm and angle of 3 deg. After the incubation process, the samples were collected following the previously described methodology for pork burgers and dry‐cured sausages.

Transepithelial electrical resistance (TEER) was measured at the beginning and the end of the culture period using a voltammeter equipped with a chopstick‐type electrode (EVOMX and STX2 electrode; World Precision Instruments, Sarasota, FL, USA) according to Okada et al. (2000). At the beginning, the TEER value of Caco‐2 was around 300 ± 49 Ω.cm^2^, indicating the formation of tight monolayers. At the end of the experiment for transepithelial transport, the TEER values were 274 ± 31 Ω.cm^2^, with no marked changes in comparison to values obtained at the beginning. This indicated that the incubation with the intestinal extract did not affect the physical properties of the Caco‐2 cells.

### 2.7 Measure of lipid content

The total fat content in both meat products and model system was determined gravimetrically with chloroform:methanol (2:1), following the method of Pérez‐Palacios et al. (2008).

### 2.8 Analysis of fatty acids

The fatty acid methyl esters (FAMEs) of extracted lipid from meat products and model system (10 mg), intestinal extracts (10 mg), and total fat obtained from the apical, basal, and membrane compartments after Caco‐2 cells incubation were prepared by acidic transesterification, as described by Sandler and Karo (2012). FAMEs were analyzed by gas chromatography (GC) using an Agilent 6890 N gas chromatograph equipped with a flame ionization detector (FID). Separation was carried out on a cyanopropyl column (ZEBRON ZB‐FAME, Phenomenex, California, USA) (20 m × 0.18 mm i.d. × 0.15 μm film thickness) with split injection (100:1). Oven temperature programming started at 150°C and it was raised 10°C/min to 180°C. This was held for 1 min and increased again at 7°C/min to 205°C which is maintained for 2 min. Injector and detector temperatures were 250°C. The carrier gas was helium at a flow rate of 2.7 mL/min. Individual FAME peaks were identified by comparison of their retention times with those of standards (Supelco 37 FAME‐mix, Merck) and quantified using tridecanoic acid (C13:0) as internal standard.

BAC was calculated based on the FA quantity after in vitro digestion with respect to the initial quantity in the sample, and BVB was calculated based on the FA in the basolateral compartment after incubation with Caco‐2 cells with respect to the initial fat quantity in the meat sample, according to Parada and Aguilera (2007).

### 2.9 Statistical analysis

In commercial pork burgers and pork dry‐cured sausages, differences in FA quantity after Caco‐2 cell incubation in apical, basal, and membrane compartments were analyzed by one‐way analyses of variance. In cooked meat model systems enriched with ω‐3 microcapsules, the effects of fat quantity, incubation time, and shake on the FA in apical, basal, and membrane compartments were analyzed by multivariate general linear model. When a significant effect (*p* < .05) was detected, paired comparisons between means were conducted using Tukey's test. The statistics were run using the program IBM SPSS Statistics v.22 (IBM Co., New York, USA).

## 3 RESULTS AND DISCUSSION

### 3.1 Bioavailability of fatty acids in meat products assessed with previous Caco‐2 cell procedures

In the present work, it was firstly tested an in vitro digestion analysis followed by a simulated intestinal culture with Caco‐2 cells based on previous works (Beguin et al., 2014; Tsuzuki, 2007; Van Breemen & Li, 2005) in pork burgers and pork dry‐cured sausages to determine the BAC and BVB of fatty acids in meat samples.

Table 1 shows the FA quantity of commercial pork burgers and dry‐cured sausages (expressed as mg FAMEs/g sample) before in vitro simulated digestion. In both meat products, monounsaturated fatty acids (MUFA) were the major family of fatty acids, the family of saturated fatty acids (SFA) in second place, and the family of polyunsaturated fatty acids (PUFA) in the lowest quantities. In pork burger, of the 16 fatty acids identified, oleic acid (17.96 mg/g sample) was the major, having higher quantities than palmitic (8.33 mg/g sample), stearic (7.66 mg/g sample), and linoleic (5.95 mg /g sample) acids, while the quantity of most fatty acids was lower than 1 mg FAMEs/g sample. This is quite in agreement with previous results in a similar meat product (López‐López et al., 2011). In dry‐cured sausage, oleic acid (111.67 mg/g sample) was also the major fatty acid, with higher quantities than palmitic (55.77 mg/g sample), linoleic (45.92 mg/g sample), stearic (45.70 mg/g sample), palmitoleic (5.77 mg/g sample), and myristic (4.09 mg/g sample) acids. In this case, minor fatty acids had quantities lower than 4 mg FAMEs/g sample. These results are in concordance with previous findings (Bañón et al., 2010; Gambacorta et al., 2009), and with the lipid profile of the ingredients of the product (previously described in Section 2.4). Besides, higher FA quantity in dry‐cured sausages than in pork burgers can be noted, which agrees with the different fat content between these meat products (37 ± 1.02 vs 16 ± 0.14%, in pork dry‐cured sausages and pork burgers, respectively).

**TABLE 1:** Bioaccessibility (BAC) of fatty acids in pork burgers and dry‐cured sausages: mg fatty acid methyl esters/g sample before and after in vitro simulated digestion.

Fatty acids	Pork burger			Pork dry‐cured sausages		
	Be	Af	BAC (%)	Be	Af	BAC (%)
C14:0	0.62 ± 0.08	0.41 ± 0.12	66.17	4.09 ± 0.57	2.93 ± 1.04	71.60
C14:1n‐5	0.11 ± 0.01	0.09 ± 0.01	79.55	0.79 ± 0.13	0.58 ± 0.08	73.41
C15:0	0.21 ± 0.02	0.16 ± 0.01	74.76	1.23 ± 0.23	1.02 ± 0.09	82.93
C15:1n‐5	0.16 ± 0.03	0.13 ± 0.01	78.27	0.75 ± 0.07	0.61 ± 0.05	81.24
C16:0	8.33 ± 0.54	5.20 ± 0.55	62.46	55.77 ± 7.64	54.91 ± 6.12	98.46
C16:1n‐7	1.04 ± 0.06	0.54 ± 0.06	52.08	5.77 ± 0.31	4.72 ± 0.50	81.82
C17:0	0.24 ± 0.02	0.11 ± 0.01	45.58	1.45 ± 0.18	1.14 ± 0.31	78.78
C17:1n‐7	0.19 ± 0.02	0.11 ± 0.01	59.85	1.15 ± 0.16	0.82 ± 0.10	71.05
C18:0	7.66 ± 1.50	5.52 ± 0.59	72.10	45.70 ± 0.92	43.06 ± 2.55	94.22
C18:1n‐9	17.96 ± 1.73	12.35 ± 3.35	69.77	111.67 ± 12.71	101.65 ± 2.69	91.03
C18:2n‐6	5.95 ± 1.05	4.49 ± 0.58	75.47	45.92 ± 5.61	42.19 ± 4.22	91.87
C18:3n‐6	0.40 ± 0.04	0.22 ± 0.03	54.91	2.61 ± 0.32	2.02 ± 0.75	77.19
C20:0	0.20 ± 0.02	0.15 ± 0.01	75.42	1.14 ± 0.06	0.97 ± 0.11	85.09
C20:1n‐7	0.36 ± 0.05	0.15 ± 0.01	40.78	2.49 ± 0.34	1.91 ± 0.57	76.66
C20:2n‐6	0.26 ± 0.02	0.12 ± 0.01	47.88	2.26 ± 0.28	1.77 ± 0.48	78.20
C20:4n‐6	0.62 ± 0.11	0.41 ± 0.03	66.67	1.38 ± 0.18	1.18 ± 0.08	85.55
∑SFA	17.68 ± 1.98	11.86 ± 1.26	67.11	111.73 ± 12.98	93.73 ± 9.59	83.89
∑MUFA	20.95 ± 1.86	13.97 ± 3.36	66.69	131.03 ± 14.70	116.85 ± 1.85	89.18
∑PUFA	7.66 ± 1.25	5.56 ± 0.63	72.62	54.30 ± 6.41	42.06 ± 5.14	77.46

*Note*: Values expressed mean ± standard deviation (SD). Myristic acid (C14:0); myristoleic acid (C14:1); pentadecanoic acid (C15:0); 5‐pentadedenoic acid (C15:1n‐5); palmitic acid (C16:0); palmitoleic acid (C16:1n‐7); margaric acid (C17:0); margaroleic acid (C17:1n‐7); stearic acid (C18:0); oleic acid (C18:1n‐9); linoleic acid (C18:2n‐6); γ‐linolenic acid (C18:3n‐6); arachidic acid (C20:0); paullinic acid (C20:1n‐7); eicosadenoic acid (C20:2n‐6); adrenic acid (C20:4n‐6); saturated fatty acids (SFA); monounsaturated fatty acids (MUFA); and polyunsaturated fatty acids (PUFA). (Be and Af) Before and after in vitro simulated digestion, respectively.

Regarding the FA quantity released after in vitro simulated digestion (expressed as mg FAMEs/g digested extract) (Table 1) in pork burger, oleic acid (12.35 mg C18:1n‐9/g digested extract) showed the major fatty acid released, showing higher quantities than palmitic (5.20 mg C16:0/g digested extract), stearic (5.52 mg C18:0/g digested extract), and linoleic (4.49 mg C18:2n‐6/g digested extract) acids, and minor fatty acids (<0.6 mg FAMEs/g sample), which is positively related to the lipid composition of undigested samples: the major FA in the product also have the highest quantities after digestion. Thus, the BAC in pork burgers ranged between 40 and 79%, with an average of 65.61%, according to previous digestion works carried out with fat, revealing 50–90% hydrolysis at the intestinal phase (Gunstone, 2001; Lairon, 2009), which is expected because lipolytic enzymes are secreted in the intestinal tract, and, consequently, most hydrolyzed lipid compounds are released at the end of this phase. A previous study on cooked and dry‐cured sausages found BAC percentages of around 34 and 71%, respectively (Solomando et al., 2020c). Thus, there seems to be a notable variability in the BAC between different meat products. As for individual FA, although it is difficult to establish a clear relationship between the BAC and FA characteristics, in general, it can be pointed out that major FA have obtained moderate–high BAC (oleic (69.77%), palmitic (62.46%), stearic (72.10%), and linoleic (75.47%) acids). This hypothesis is difficult to contrast since there are no similar previous works evaluating the effect of FA composition on in vitro BAC. Besides, contradictory results have been found about the influence of the feed composition on lipid digestibility in animals (Annor et al., 2015; Tancharoenrat et al., 2014). The stereospecific distribution of FA in triacylglycerols can be behind the BAC of FA in meat products since previous works carried out with animals have reported a major absorption of FA from the sn‐2 position of dietary triglycerides (Hunter, 2001). In pork samples, the ratio SFA:UFA is 1:3 for the *sn*1,3 positions and 3:1 for the *sn*2 position (Di Luccia et al., 2003). These statements would suppose a major BAC of SFA. However, this is not clearly observed in pork burger results of this experiment.

The BAC in dry‐cured sausage ranged between 71% and 98%, with an average of 84.65%, which is higher than BAC found in pork burgers. It is also noted that major FA allowed the highest BAC in dry‐cured sausages, which pointed out that the FA quantity and its BAC are positively correlated in this product. However, in the work of Solomando et al. (2020c), with cooked and dry‐cured sausages, the fat and FA release at the end of the intestinal phase was higher in cooked sausages, with lower fat quantity, than in dry‐cured sausages. This was ascribed to the presence of areas with low exposure to lipolytic enzymes, such as solid fat and large‐size fat globules. Wang et al. (2022) reported that the existence of lactic acid bacteria in dry‐cured meat products increased lipolytic enzymes that further improve digestibility. As explained, the dry‐cured sausages of this study were purchased in a local market, so they should have been added with lactic acid bacteria, which were not added to the dry‐cured sausages elaborated by Solomando et al. (2020c). Thus, the different findings in FA release at the end of the intestinal phase can be related to the differences in the product matrix, ingredients, and processing.

These findings from in vitro digestion of pork burgers and dry‐cured sausages point out that the lipid content, the FA quantity in the product, and the FA quantity released at the end of the intestinal phase are positively correlated, while the BAC seems to be more correlated with the lipid content than with the FA quantity. In addition, these results ensure that an intestinal extract is subjected to be incubated with Caco‐2 cells.

Thus, continuing with the first stage, the intestinal extract was incubated with Caco‐2 cells under conditions based on previous studies (Beguin et al., 2014; Tsuzuki, 2007; Van Breemen & Li, 2005). Table 2 shows the FA quantity (expressed as mg FAMES/mL of incubated digested solution) measured in the three compartments of the Transwell: apical solution (AS), cell monolayer (CM), and basal solution (BS). In general, the sums of SFA, MUFA, and PUFA showed significant differences in both meat products studied, finding higher quantities in AS and BS compartments than in CM. In the case of individual FA, of the 16 fatty acids previously quantified in pork burgers before and after simulated in vitro digestion, only 5 of them were identified after experimental transport with Caco‐2 cells, and the rest showed quantities lower than the limit of quantification (LOQ) and all the identified FA with significant differences. The quantities of palmitic, palmitoleic, and oleic acids were higher in the AS and BS compartments than in CM; in the case of stearic and linoleic acids, the highest quantities were found in AS followed in decreasing order by BS and CM. As for BVB, the average percentage was 6.16%. Of all the FA identified, palmitoleic acid showed the highest BVB (15.94%), being higher than palmitic acid (5.70%), and being the BVB for the rest of fatty acids lower than 4. In dry‐cured sausages, of the 16 FA previously quantified before and after simulated in vitro digestion, only 11 of them were identified after experiment transport with Caco‐2 cells, the rest showing quantities <LOQ. Higher quantities of palmitic, stearic, oleic, linoleic, and γ‐linolenic acids were found in AS and BS than in CM. As for the BVB in dry‐cured sausages, the average percentage was 6.15%. Of all the fatty acids identified, arachidic acid showed the highest percentage (9.49%), with margaroleic, margaric, myristic, palmitoleic, palmitic, paullinic, linoleic, and oleic acid showing lower BVB (8.76%, 7.94%, 7.11%, 6.80%, 5.30%, 5.27%, 4.99%, and 4.16%, respectively).

**TABLE 2:** Bioavailability (BVB) and fatty acid quantity (expressed as mg FAMEs/mL incubated digested solution) in the apical solution (AS), cell monolayer (CM), and basal solution (BS) after transport experiments for 4 h with Caco‐2 cells with intestinal extracts from in vitro digestion of pork burgers and dry‐cured sausages.

Fatty acids	Pork burger			*p*	BVB (%)	Pork dry‐cured sausages			*p*	BVB (%)
	AS	CM	BS			AS	CM	BS		
C14:0	<LOQ	<LOQ	<LOQ	–	–	0.07 ± 0.01	0.03 ± 0.00	0.05 ± 0.02	ns	7.11
C14:1n–5	<LOQ	<LOQ	<LOQ	–	–	<LOQ	<LOQ	<LOQ	–	–
C15:0	<LOQ	<LOQ	<LOQ	–	–	<LOQ	<LOQ	<LOQ	–	–
C15:1n‐5	<LOQ	<LOQ	<LOQ	–	–	<LOQ	<LOQ	<LOQ	–	–
C16:0	0.09^a^ ± 0.02	0.04^b^ ± 0.01	0.07^a^ ± 0.01	*	5.70	0.76^a^ ± 0.08	0.19^b^ ± 0.01	0.61^a^ ± 0.19	**	5.30
C16:1n‐7	0.01^a^ ± 0.00	<LOQ ^b^	0.01^a^ ± 0.00	*	15.94	0.08 ± 0.00	0.03 ± 0.00	0.08 ± 0.06	ns	6.80
C17:0	<LOQ	<LOQ	<LOQ	–	–	0.02 ± 0.00	0.02 ± 0.01	0.02 ± 0.01	ns	7.94
C17:1n‐7	<LOQ	<LOQ	<LOQ	–	–	0.02 ± 0.00	0.02 ± 0.00	0.02 ± 0.00	ns	8.76
C18:0	0.07^a^ ± 0.01	0.02^c^ ± 0.00	0.04^b^ ± 0.00	**	3.27	0.34^a^ ± 0.02	0.09^b^ ± 0.00	0.26^a^ ± 0.09	***	2.73
C18:1n‐9	0.10^a^ ± 0.03	0.03^b^ ± 0.02	0.09^a^ ± 0.02	*	3.06	1.44^a^ ± 0.01	0.28^b^ ± 0.09	1.05^a^ ± 0.31	***	4.16
C18:2n‐6	0.06^a^ ± 0.02	0.01^c^ ± 0.00	0.03^b^ ± 0.00	**	2.87	0.63^a^ ± 0.04	0.14^b^ ± 0.03	0.43^a^ ± 0.22	**	4.99
C18:3n‐6	<LOQ	<LOQ	<LOQ	–	–	<LOQ	<LOQ	<LOQ	–	–
C20:0	<LOQ	<LOQ	<LOQ	–	–	0.03 ± 0.00	0.02 ± 0.00	0.03 ± 0.01	ns	9.49
C20:1n‐7	<LOQ	<LOQ	<LOQ	–	–	0.05^a^ ± 0.00	0.01^b^ ± 0.00	0.02^b^ ± 0.01	**	5.27
C20:2n‐6	<LOQ	<LOQ	<LOQ	–	–	0.01 ± 0.00	0.02 ± 0.01	0.01 ± 0.01	ns	3.06
C20:4n‐6	<LOQ	<LOQ	<LOQ	–	–	<LOQ	<LOQ	<LOQ	–	–
∑SFA	0.15^a^ ± 0.04	0.06^b^ ± 0.01	0.12^a^ ± 0.01	**	4.02	1.22^a^ ± 0.07	0.35^b^ ± 0.07	0.97^a^ ± 0.32	***	4.18
∑MUFA	0.11^a^ ± 0.05	0.03^b^ ± 0.02	0.12^a^ ± 0.02	**	3.32	1.60^a^ ± 0.01	0.34^b^ ± 0.08	1.17^a^ ± 0.44	**	4.04
∑PUFA	0.06^a^ ± 0.02	0.01^c^ ± 0.00^c^	0.03^b^ ± 0.00	**	2.32	0.72^a^ ± 0.04	0.19^b^ ± 0.03	0.52^a^ ± 0.26	**	4.98

*Note*: Values expressed mean ± standard deviation (SD). Significance level in ANOVA; *: *p* < .05; **:*p* < .01; ***:*p* < .001; ns: not significant. Bars with different letters (a, b, c) within the same fatty acid show significant differences (*p* < .05) in post‐hoc analysis due to compartment. Myristic acid (C14:0); myristoleic acid (C14:1); pentadecanoic acid (C15:0); 5‐pentadedenoic acid (C15:1n‐5); palmitic acid (C16:0); palmitoleic acid (C16:1n‐7); margaric acid (C17:0); margaroleic acid (C17:1n‐7); stearic acid (C18:0); oleic acid (C18:1n‐9); linoleic acid (C18:2n‐6); γ‐linolenic acid (C18:3n‐6); arachidic acid (C20:0); paullinic acid (C20:1n‐7); eicosadenoic acid (C20:2n‐6); adrenic acid (C20:4n‐6); saturated fatty acids (SFA); monounsaturated fatty acids (MUFA); and polyunsaturated fatty acids (PUFA).

Abbreviation: LOQ, limit of quantification.

These results evidenced an efficient FA transport during the culture with Caco‐2 cells. However, it is important to note the impossibility of determining minor fatty acids. This is specially marked in the sample with lower lipid content, which may be an influencing parameter BVB. In fact, Salvia‐Trujillo et al. (2017) have indicated high variability in the BVB of the lipid compounds (10 to 80%) depending on the characteristics of the food matrix. The incubation time may also affect the fatty transport through Caco‐2 cells; although the Caco‐2 cells differentiate into mature cells similar to those from the epithelium of the small intestine, they also have characteristics that differ from native enterocytes (Ho et al., 2002), with activity levels of monoacylglycerol acyltransferase around 10% of those found in mature villus (Ho & Storch, 2001). Besides, during the incubation process, the chemical composition and the structure of the compounds present in the extract change, which could influence the solubility, stability, and availability of the compounds for absorption (Carbonell‐Capella et al., 2014). In this sense, previous works have suggested that longer incubation times could increase the release and solubilization of the compounds contained in the incubated extract, being able to increase the average BVB percentage. Nevertheless, prolonged incubation times could lead to degradation or modifications of chemical forms, leading to decreases in total BVB (Garrett et al., 2000). Thus, there is no consensus about the incubation among researchers; that is, in Park and Carr (2013), a mixture of palmitic, stearic, oleic, linoleic, γ‐linolenic, and eicosapentaenoic acids was incubated for 18 h; in the study of Ho et al. (2002) with palmitic and oleic acid, 2, 6, and 24 h were applied; Vors et al. (2012) tested 4, 8 and 12 h for incubating the gastrointestinal lipolysis medium of O/W emulsions; and Tsuzuki (2007) incubated lipid metabolites from mixed micelles for 2 h. In addition, Kono et al. (2016) have hypothesized the influence of the shaking during the intestinal FA absorption, since it could allow breaking down of large lipid droplets into smaller droplets, which could increase the surface area available for absorption by the differentiated cell monolayer during the transit process.

Therefore, the next step of this work was to determine the influence of incubation conditions (fat quantity, incubation time, and shake) of the intestinal extract with a monolayer of polarized Caco‐2 on the FA transport.

### 3.2 Effect of incubation conditions of intestinal extracts with Caco‐2 cell on transport and bioavailability of fatty acids in meat samples

Table 3 shows the FA profile before and after in vitro digestion assay of cooked meat model system enriched with multilayer fish oil microcapsules. MUFA showed the highest quantities, followed in decreasing order by SFA and PUFA with the lowest quantities. Of the 22 FA identified, oleic acid (27.68 mg/g sample) showed the highest quantity, followed in decreasing order by palmitic (18.60 mg/g sample), stearic (7.63 mg/g sample), and linoleic (5.72 mg/g sample) acids, with the rest of fatty acids showing concentrations lower than 5 mg FAMEs/g sample. It is worth mentioning that the sum of EPA + DHA was 96 mg EPA + DHA/100 g, which exceeded the minimum level established by the legislation to label a food as “high in ω‐3 fatty acids” (EU, 2010). So, the type of fish oil microcapsules used in this study was achieved to enrich in ω‐3 PUFA, as desired. These results reflect the FA composition of the ingredients used in the elaboration of the model system, mainly with pork meat, pork fat, and fish oil microcapsules as a source of ω‐3 fatty acids, and are in agreement with previous studies in cooked pork sausages (Juárez et al., 2009; Yilmaz et al., 2002).

**TABLE 3:** Bioaccessibility (BAC) of fatty acids on cooked model system enriched with multilayered fish oil microcapsules: mg fatty acid methyl esters/g sample before (Be) and after (Af) in vitro simulated digestion.

Fatty acids	Be	Af	BAC (%)
C10:0	0.20 ± 0.02	0.08 ± 0.02	39.45
C11:0	0.09 ± 0.01	<LOQ	0.00
C12:0	0.20 ± 0.02	0.11 ± 0.01	54.73
C14:0	1.25 ± 0.15	1.03 ± 0.06	83.05
C15:0	0.07 ± 0.01	<LOQ	0.00
C15:1n‐5	0.05 ± 0.03	<LOQ	0.00
C16:0	18.60 ± 2.44	12.05 ± 0.83	64.77
C16:1n‐7	3.18 ± 0.51	1.82 ± 0.11	57.08
C17:0	0.19 ± 0.04	0.18 ± 0.02	95.41
C17:1n‐7	0.19 ± 0.02	0.17 ± 0.04	92.66
C18:0	7.63 ± 1.34	6.70 ± 0.49	87.73
C18:1n‐9	27.68 ± 3.63	18.11 ± 1.26	65.43
C18:2n‐6	5.72 ± 0.76	3.68 ± 0.23	64.29
C18:3n‐6	0.04 ± 0.04	<LOQ	0.00
C18:3n‐3	0.37 ± 0.04	0.29 ± 0.03	76.78
C20:1n‐7	0.60 ± 0.16	0.59 ± 0.09	99.05
C20:2n‐6	0.27 ± 0.05	0.27 ± 0.06	99.79
C20:4n‐6	0.34 ± 0.12	0.26 ± 0.02	77.85
C22:1n‐11	0.22 ± 0.03	<LOQ	0.00
C20:5n‐3 EPA	0.13 ± 0.16	0.13 ± 0.03	99.79
C24:1n‐9	0.37 ± 0.08	0.24 ± 0.05	64.94
C22:6n‐3 DHA	0.83 ± 0.24	0.65 ± 0.08	78.31
∑SFA	28.23 ± 4.03	20.14 ± 1.43	71.37
∑MUFA	32.38 ± 4.48	20.93 ± 1.55	64.64
∑PUFA	8.54 ± 1.51	5.97 ± 0.44	69.95

*Note*: Values expressed mean ± standard deviation (SD); Capric acid (C10:0); undecyl acid (C11:0); lauric acid (C12:0); myristic acid (C14:0); pentadecanoic acid (C15:0); 5‐pentadedenoic acid (C15:1n‐5); palmitic acid (C16:0); palmitoleic acid (C16:1n‐7); margaric acid (C17:0); margaroleic acid (C17:1n‐7); stearic acid (C18:0); oleic acid (C18:1n‐9); linoleic acid (C18:2n‐6); γ‐linolenic acid (C18:3n‐6); α‐linolenic acid (C18:3n‐3); paullinic acid (C20:1n‐7); eicosadenoic acid (C20:2n‐6); adrenic acid (C20:4n‐6); ketoleic acid (C22:1n‐11); eicosapentaenoic acid (C20:5n‐3 EPA); nervonic acid (C24:1n‐9); docosahexaenoic acid (C22:6n‐3 DHA); saturated fatty acids (SFA); monounsaturated fatty acids (MUFA); and polyunsaturated fatty acids (PUFA). (Be and Af) Before and after in vitro simulated digestion, respectively.

Abbreviation: LOQ, limit of quantification.

Regarding the individual FA released after in vitro simulated digestion (expressed as mg FAMEs/g digested extract), oleic acid showed the highest quantity (18.11 mg/g digested extract), followed in decreasing order by palmitic (12.05 mg/g digested extract) and stearic (6.70 mg/g digested extract) acids, with the rest of the FA showing quantities lower than 6 mg FAMEs/g digested extract. Again, it is noted the higher released in the major FA.

Results on BAC in cooked model systems enriched with multilayered fish oil microcapsules showed a high variability, from 57.73 to 99.79%, with most FA showing BAC percentages higher than 75%. It is also noted that major FA (oleic and palmitic acids) obtained moderate BAC (65.43 and 64.77%, respectively), while the highest percentage was found in minor FA, including added EPA and DHA (99.79 and 78.31%, respectively). These results are more in concordance with those found in the burger samples of the present work than with dry‐cured sausages BAC and reinforce the difficulty of correlating the FA quantity and BAC, as previously indicated. The high BAC of EPA and DHA (added through the fish oil microcapsules) indicates that the wall of the microcapsules (made of a chitosan–maltodextrin multilayer structure) may be resistant to gastric digestion and allows the fatty release at intestinal level. Yongsheng et al. (2008) have reported that chitosan provides protection against acid conditions. Moreover, in the present work, the electrostatic attraction between the lecithin layer (negatively charged) with maltodextrin and the chitosan layer (positively charged) can also contribute to the explained results. At the gastric phase, with acidic pH, the chitosan is charged positively (Hur et al., 2011; McClements & Li, 2010), there is a high electrostatic attraction with the lecithin, and consequently, the fish oil is difficult to release. However, at the intestinal phase, with pH between 6 and 7.5, the chitosan losses it positive charge and the electrostatic interaction diminishes, which may favor the fish oil release. Guaranteeing a high BAC of EPA and DHA is a key stage in ω‐3‐enriched products to investigate their epithelial transport.

**FIGURE 2:**
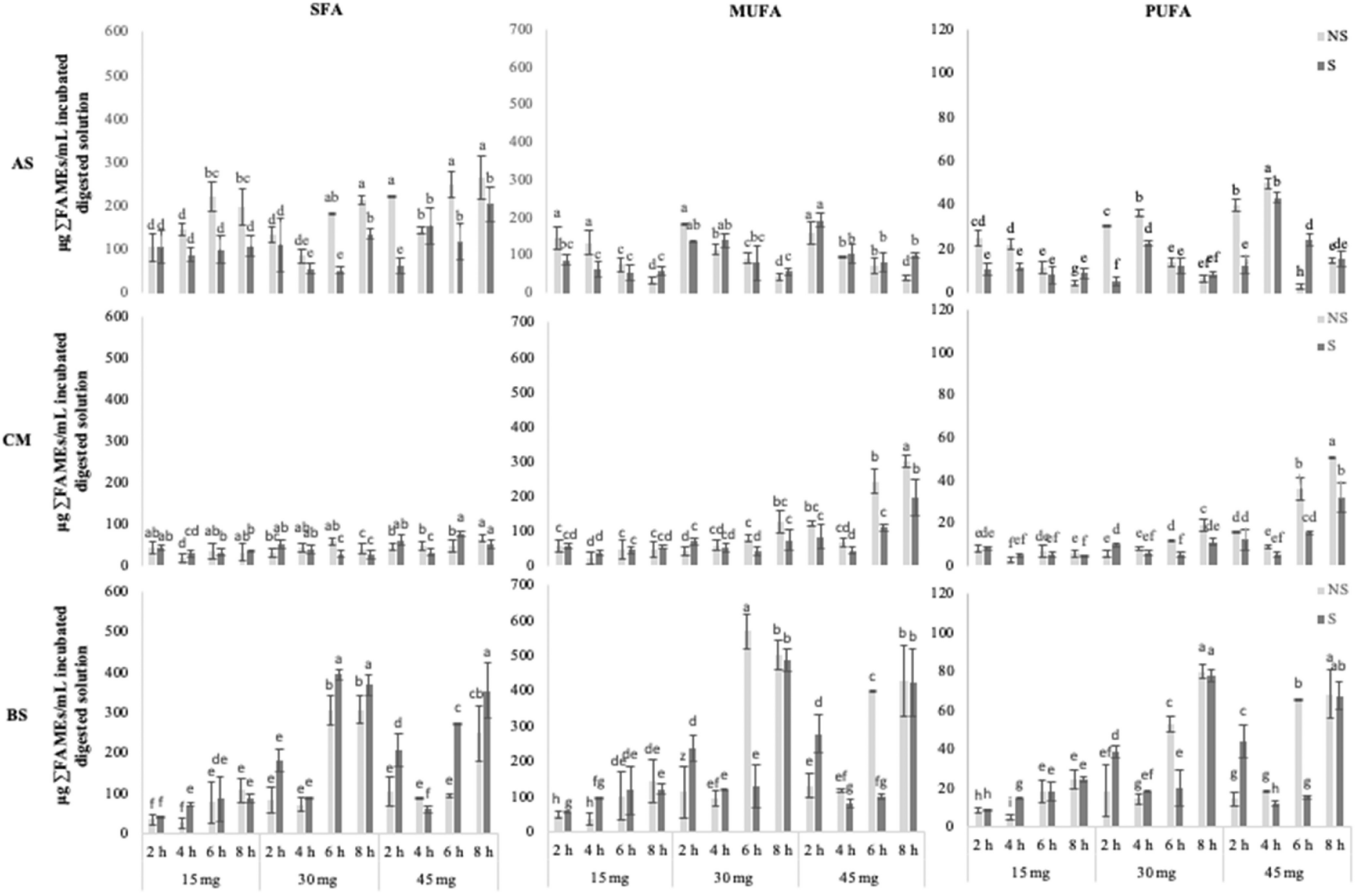
Quantity of saturated (SFA), monounsaturated (MUFA), and polyunsaturated fatty acids (PUFA) expressed as μg ∑FAMEs/mL incubated digested solution after transport experiment with Caco‐2 cells under different experimental conditions (fat quantity (15, 30, and 45 mg of fat/mL intestinal extract), incubation time (2, 4, 6, and 8 h), and shake (without light gray; with dark gray) in the apical solution (AS)), cell monolayer (CM), and basal solution (BS). Different letters indicated significant difference between the analyzed data.

Once the fatty acids BAC was assured, especially that from EPA and DHA, the intestinal extract from cooked meat model system enriched with multilayer fish oil microcapsules was incubated with Caco‐2 cells under different conditions (fat quantity, incubation time, and shake). Figure 2 shows the sums of SFA (C10:0, C12:0, C14:0, C15:0, C16:0, C17:0, and C18:0), MUFA (C15:1n‐5, C16:1n‐7, C17:1n‐7, C18:1n‐9, C20:1n‐9, C22:1n‐11, and C24:1n‐9), and PUFA (C18:2n‐6, C18:3n‐3, C20:2n‐6, C20:4n‐6, C20:5n‐3, and C22:6n‐3) (expressed as mg FAMEs/mL incubated digested solution) at the end of the Caco‐2 cell incubation in AS, CM, and BS compartments of the transwell. It is worth mentioning the detection and quantification of most FA in the Transwell compartments, while in the first trial, most of the minor FA were under the limit of quantification in AS, CM, and BS compartments. This fact is quite important when the target is in minor fatty acids, such as EPA and DHA.

**FIGURE 3:**
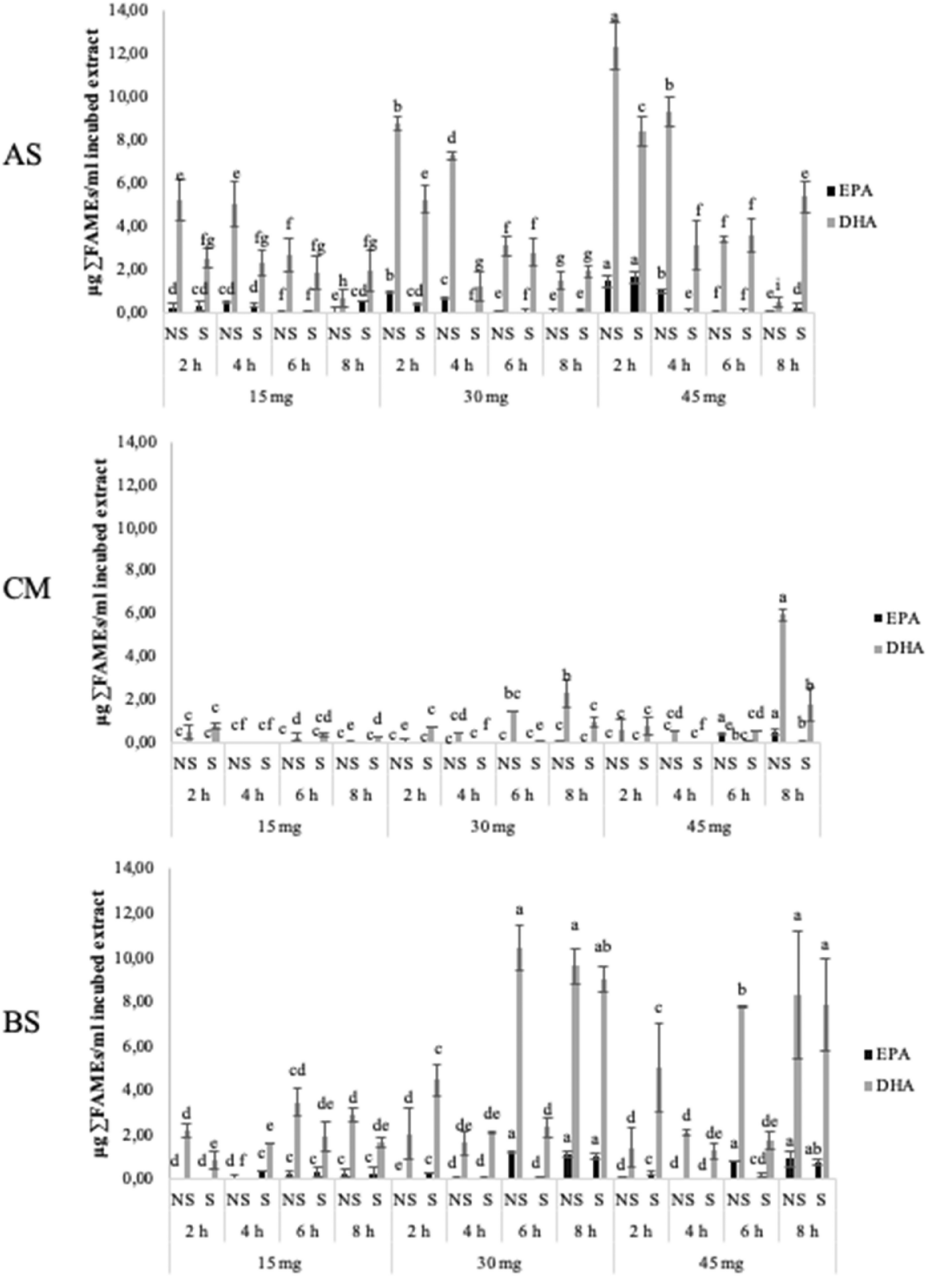
Quantity of eicosapentaenoic (EPA) and docosahexaenoic (DHA) fatty acids expressed as μg ∑FAMEs/mL incubated digested solution after transport experiment with Caco‐2 cells under different experimental conditions: fat quantity (15, 30, and 45 mg of fat/mL intestinal extract), incubation time (2, 4, 6, and 8 h), and shake (without light gray; with dark gray) in the apical solution (AS), cell monolayer (CM), and basal solution (BS). Different letters indicated significant difference between the analyzed data.

The statistical results have shown significance (*p* < .05) of the three variables, individually (fat quantity, incubation time, and shake: *p*C, *p*T, and *p*S, respectively) as well as in combination (*p*CxTxS), and there are also significant differences in the FA quantities among compartment: AS versus CM versus BS (*p* < .001). In general, the highest quantities of the sum of SFA, MUFA, and PUFA (Figure 2) and of EPA and DHA (Figure 3) were observed in BS, followed, in decreasing order, by AS and CM. This is especially noted with higher fat quantity in the intestinal extract and longer incubation time. It also points out an appropriate intestinal transport of lipids, since triacylglycerol is hydrolyzed by pancreatic lipase into free FA and *sn*‐2‐monoacylglycerol, which are absorbed across the apical surface of enterocyte, reincorporated into TG, and secreted in the chylomicrons by the basolateral membrane (Iqbal & Hussain, 2009).

Regarding the effect of the fat quantity in the intestinal extract on the FA transport, different behaviors depending on the FA type can be seen (Figure 2). In the AS compartment, SFA and PUFA increased with the fat quantity, especially with shorter incubation times, and MUFA remained quite constant. In the CM compartment, SFA did not show marked changes, and MUFA and PUFA quantities were higher when incubating 45 mg of fat in comparison to 15 and 30 mg, this effect being more noticeable at longer incubation times. In the BS compartment, SFA, MUFA, and PUFA showed a similar trend, finding, in general, higher quantities when incubating 30 mg in comparison to 15 and 45 mg. Again, this effect was more noticeable at longer incubation times. In the case of EPA and DHA (Figure 3), they showed a similar trend than PUFA. Thus, in general, the highest FA quantities in BS compartment were found when using 30 mg of fat content in the intestinal extract, followed by 45 mg, while 15 mg obtained the lowest quantities. This agrees with the research of Murota and Storch (2005) and Park and Carr (2013), who observed a linear relationship between the FA uptake and their concentration in the incubation medium. Studies on humans have reported a positive correlation between amount of fat in food and absorption of ω‐3 PUFA (Schuchardt et al., 2014) which is quite in concordance with the results of the present work. On the other hand, the increase in FA quantities in AS and CM compartments as the fat quantity increases may be explained by the existence of passive diffusion, being free FA and *sn*‐2‐monoacylglycerol also taken up across the BS surface (Ho & Storch, 2001), especially when lipid concentrations are high.

As for the incubation time effect (Figures 2 and 3) in AS, the quantities of SFA were higher at shorter times (2 and 4 h) than at longer ones (6 and 8 h), and the contrary effect was observed for MUFA and PUFA, with higher quantities at shorter times. In CM, the most notable differences were found for MUFA and PUFA when using 45 mg of fat quantities in the intestinal extract, having higher quantities at longer times. In BS, for SFA, MUFA, and PUFA, higher quantities were found at longer times. EPA and DHA behaved similarly than PUFA. These findings pointed out a clear relationship between the transepithelial lipid transport and the incubation time, in agreement with other authors, who observed a significantly higher net uptake of palmitic and oleic acids in the BS compartment when lipid extracts were incubated for longer periods (6 and 24 h) compared to shorter incubation periods (2 h) (Ho et al., 2002). Due to the high MUFA and PUFA quantities in CM compartment when incubating 45 mg of fat, the adjustment between incubation time and fat quantity in the intestinal extract seems to be needed. Besides, the coexistence of active and passive transport has been also revealed in evaluating the influence of the incubation time because of the higher SFA quantities in AS at 6 and 8 h of incubation. This fact indicates the goodness of the present procedure for Caco‐2 cell culture, allowing a polarized monolayer with access to AS, CM, and BS compartments for intestinal lipid absorption studies.

In relation to the shake effect (Figures 2 and 3), similar FA quantities have been found in shaken and not shaken samples in most cases in CM and BS compartments, However, this effect has been more noticeable in the AS compartment for SFA, obtaining lower FA quantities when not shaking. It should be indicated that shaking Caco‐2 cell culture enhances the lipid active transport of SFA from AS to BS, suggesting the need to keep shaking the intestinal extracts during incubation with Caco‐2 cells. This effect should be complementary to the bile acids’ action to form mixed micelles in an aqueous environment and improve the transport vehicle to deliver fatty acids from the apical to the basolateral membrane. Besides, the results of the present study supported the hypothesis proposed by Kono et al. (2016) who postulated that constant agitation of the intestinal extracts during the incubation period could lead to a transformation of large droplets into smaller micelles to increase the contact surface and therefore the area available for its uptake by Caco‐2 cells. Other previous studies in Caco‐2 cells have not indicated shaking the Caco‐2 cell monolayer to facilitate the access of linolenic, eicosapentaenoic (Park & Carr, 2013), arachidonic, and docosahexaenoic acids (Field et al., 2002), also in concordance with the results obtained in the present work.

**FIGURE 4:**
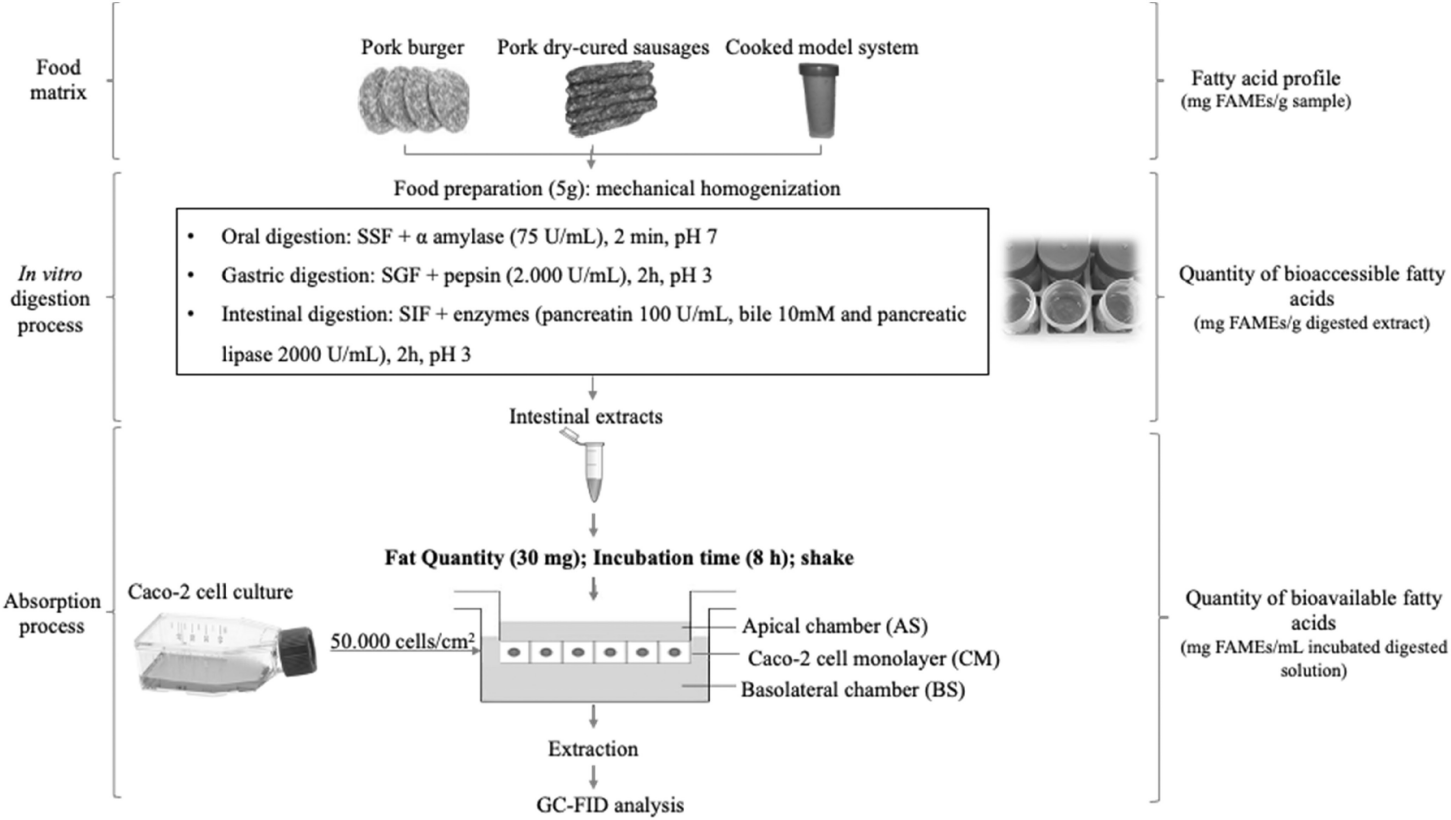
Summary of optimum digestion and culture conditions with Caco‐2 cells in different meat matrices to determine bioaccessibility and bioavailability of fatty acids. SSF, SGF, and SIF represent simulated salivary, gastric, and intestinal fluids, respectively. AS, CM, and BS represent apical solution, cell monolayer, and basal solution, respectively.

Giving a step forward, the BVB of SFA, MUFA, PUFA, EPA, and DHA as affected by fat quantity in the intestinal extract, incubation time, and shaking was calculated (Table 4). As expected, the obtained results are quite in concordance with the FA transport, finding, in general, the highest BVB with intermediate fat quantity (30 mg), longer incubation times (8 h), and shaking. Figure 4 shows a summary of the proposed conditions for in vitro digestion and subsequent culture with Caco‐2 cells to determine BAC and BVB of fatty acids in meat matrix.

**TABLE 4:** Bioavailability (BVB) of fatty acids in intestinal extract from cooked meat model enriched with fish oil microcapsules incubated with Caco‐2 cells under different experimental conditions: fat quantity (15, 30, and 45 mg of fat/mL intestinal extract), incubation time (2, 4, 6, and 8 h), and shake (NS and with S).

Fat quantity (mg)	Incubation time (h)	Not shaken or shaken (NS or S)	∑SFA	∑MUFA	∑PUFA	EPA	DHA
15	2	NS	1.03	1.21	0.80	0.17	1.06
		S	1.20	1.40	0.81	0.17	0.42
	4	NS	0.78	0.93	0.47	0.15	0.00
		S	2.09	2.41	1.38	2.13	0.79
	6	NS	2.27	2.55	1.70	1.50	1.68
		S	2.50	2.96	1.74	1.92	0.95
	8	NS	3.12	3.61	2.33	1.65	1.41
		S	2.56	3.04	2.30	1.53	0.80
30	2	NS	2.45	2.82	1.76	0.00	1.00
		S	5.22	5.92	3.66	1.68	2.17
	4	NS	2.09	2.36	1.35	0.17	0.80
		S	2.59	2.97	1.75	0.54	1.04
	6	NS	8.76	14.19	5.01	7.68	5.08
		S	11.26	3.22	1.87	0.14	1.14
	8	NS	8.81	12.48	7.55	6.86	4.65
		S	10.54	12.11	7.35	6.40	4.39
45	2	NS	2.98	3.27	1.36	0.45	0.69
		S	5.98	6.94	4.13	1.78	2.43
	4	NS	2.50	2.96	1.74	0.28	1.01
		S	1.76	2.03	1.13	0.38	0.62
	6	NS	2.75	9.91	6.20	5.19	3.77
		S	7.80	6.49	5.45	5.66	3.05
	8	NS	7.14	10.67	6.45	5.76	4.03
		S	10.16	10.56	6.37	4.82	3.82

Abbreviations: DHA, docosahexaenoic acid; EPA, eicosapentaenoic acid; MUFA, monounsaturated fatty acids; PUFA, polyunsaturated fatty acids; SFA, Saturated fatty acids.

The capability of this methodology to quantify EPA and DHA in AS, CM, and BS compartments is remarkable, contributing to the need to investigate systematically the bioavailability of ω‐3 PUFA, as demanded by Schuchardt et al. (2014) and making it easier to compare among different studies. In fact, in a recent study focused on evaluating the BAC and BVB of ω‐3 fatty acids by using in vitro digestion and cell model with Caco‐2 and HT29‐MTX E12 lines, there was no FA transport to the BS compartment, which authors ascribed to the influence of the ω‐3 fatty acids incorporated into the cells on the membrane permeability (Salsinha et al., 2023). Consequently, it was not possible to calculate the BVB of ω‐3 fatty acids in that work. In response to this limitation, the complete protocol proposed in the present study provides the BAC and BVB of fatty acids. Although it has been specifically designed for meat products enriched in ω‐3 fatty acids, it should be appropriate for most fatty acids in different food matrices.

## 4 CONCLUSIONS

This study successfully achieves a combined protocol to assess the bioaccessibility and bioavailability of fatty acids in meat samples. Influence of incubation condition of intestinal extracts from in vitro static digestion with differentiated Caco‐2 cell monolayer has been evaluated. The fat quantity in the intestinal extract and the incubation time significantly influenced the transport and bioavailability of most fatty acids. The effect of shaking was not so marked but it seems to improve the bioavailability of SFA. Moreover, the relationship between these two influencing factors was noted. The use of intestinal extracts with 30 mg of fat, longer incubation times (8 h), and shaking were shown to be the most suitable conditions, achieving active and passive fatty acid transport without compromising the integrity of the Caco‐2 cell monolayer. The accurate results obtained for major and minor fatty acids, specifically EPA and DHA, are remarkable due to the interest in these bioactive compounds.

## AUTHOR CONTRIBUTIONS

**Juan Carlos Solomando:** Data curation (equal); formal analysis (equal); investigation (equal); validation (equal); writing – original draft (equal). **Teresa Antequera:** Methodology (equal); resources (equal); writing – review and editing (equal). **Matías Estaras:** Formal analysis (equal); investigation (equal); validation (equal). **Antonio González:** Formal analysis (equal); investigation (equal); validation (equal). **Trinidad Perez‐Palacios:** Conceptualization (equal); funding acquisition (equal); project administration (equal); supervision (equal); writing – review and editing (equal).

## FUNDING INFORMATION

Ministerio de Asuntos Económicos y Transformación Digital, Gobierno de España. Agencia Estatal de Investigación AGL2016‐73260‐JIN.

## CONFLICT OF INTEREST STATEMENT

The authors have no conflicts of interest to declare. All co‐authors have seen and agree with the contents of the manuscript and there is no financial interest to report. We certify that the submission is original work and is not under review at any other publication.

## Data Availability

Data are available on request from the authors.
